# Local delivery of linezolid in the treatment of complex orthopedic bone and joint infections in patients with vancomycin allergy: a case series

**DOI:** 10.5194/jbji-9-121-2024

**Published:** 2024-03-22

**Authors:** Abhijith Annasamudram, Aja Janyavula, Ahmed H. Elhessy, Raj Krishna Shrestha, Martin Gesheff, Janet D. Conway

**Affiliations:** International Center for Limb Lengthening, Rubin Institute for Advanced Orthopedics, Sinai Hospital of Baltimore, Baltimore, Maryland, USA

## Abstract

**Introduction**: Osteomyelitis is a challenging bone infection associated with ischemia, trauma, or various surgical procedures (e.g., joint reconstruction). Treatment involves eradicating infected bone and soft tissue, local antibiotic delivery, and a 6-week course of antibiotics. Methicillin-resistant *Staphylococcus aureus* (MRSA) infections are common, and vancomycin is the standard treatment, but alternatives like linezolid are needed in vancomycin-resistant and vancomycin-allergic patients. **Methods**: A retrospective chart review was conducted on patients treated by the senior author between 2013 and 2021. The study included patients who received local delivery of linezolid for bone and/or joint infection with documented evidence of vancomycin allergy. Patient demographics, surgical details, linezolid delivery method, and outcomes were recorded. Clinical outcomes and subsequent procedures leading to infection eradication were documented. **Results**: A total of 13 patients were treated with linezolid-antibiotic-laden spacers with polymethyl methacrylate (PMMA) carrier. Nine patients were successfully treated using limb-salvage techniques and were still infection-free after a mean follow-up of 55.5 months. **Conclusions**: Linezolid-loaded bone cement is an option for managing chronic bone and joint infections, particularly MRSA, in patients with vancomycin allergy.

## 1 Introduction

Osteomyelitis (OM) poses a significant challenge in orthopedics, affecting 1 in 675 US hospital admissions annually (Nichol et al., 2016). Linked to ischemia, trauma, or surgical procedures, OM can be caused by various microorganisms (Lew and Waldvogel, 1997; Waldvogel et al., 1970). Standard treatment involves eradicating infected bone tissue, local antibiotic delivery, and a 6-week intravenous antibiotic course (Wassif et al., 2021). Methicillin-resistant *Staphylococcus aureus* (MRSA) contributes to over one-third of OM cases (Crawford et al., 2012; Darley and MacGowan, 2004; Hatzenbuehler and Pulling, 2011), and vancomycin is the standard antibiotic therapy. With the increase in vancomycin resistance and allergy there is a need for alternative treatments. Linezolid is an effective alternative, with documented studies indicating its systemic use for treating resistant staphylococcal infections (Bassetti et al., 2005; Bounthavong and Hsu, 2010). In vitro studies have also demonstrated linezolid's effectiveness as a bactericidal antibiotic controlling biofilm, making it valuable for chronic OM and fracture-related infections. Comparatively, linezolid shows similar drug elusion properties to common antibiotics in bone cement applications (Jagadale et al., 2019; Metsemakers et al., 2020; Sader et al., 2010). This present study documents the effective clinical use of local linezolid delivery in a bone cement carrier to treat vancomycin-allergic patients with OM.

## 2 Materials and methods

A retrospective study analyzed patients treated between 2013 and 2021 at our institution by the senior author for bone or joint infections. Exempted from institutional review board review, 13 eligible patients were studied who had vancomycin allergy, received linezolid via polymethyl methacrylate (PMMA) spacers, and had post-surgery records. Data encompassed demographics, linezolid dose, delivery method, other antibiotics, comorbidities, and adverse reactions. Clinical outcomes, subsequent procedures, and infection resolution were documented. The selected patients were followed until 2023.

Infection was defined as per the musculoskeletal infection society recommendations: “Presence of a draining sinus, presence of an abscess clinically or erythema, radiographic presence of osteolysis or changes in the bone on MRI consistent with infection, positive intraoperative biopsies and elevated serologic markers like C-reactive protein and erythrocyte sedimentation rate” (Parvizi et al., 2018). This criterion has been adopted for this study. Treatment followed culture-based antibiotics. Infection resolution was assessed post-op via C-reactive protein, erythrocyte sedimentation rate, clinical, and radiological methods.

### Bone cementing technique

Following a method previously published by the senior author (Pargas et al., 2022), antibiotics were delivered using calcium sulfate (CS), PMMA, or a combination of the two. PALACOS bone cement (Zimmer Biomet, Warsaw, IN, USA) was utilized, with spacers and coatings tailored to individual cases. Linezolid, mixed exclusively with PMMA, and tobramycin, mixed with both PMMA and CS, were used in the PALACOS bone cement. Linezolid tablets (600 mg) were used in an off-label fashion after obtaining consent from the patients. This off-label use does not need emergency approval and was clarified to the Institutional Review Board. Analysis in Excel (Microsoft, Redmond, WA, USA) involved summarizing baseline demographics and calculating descriptive statistics for continuous and categorical data (Pargas et al., 2022).

## 3 Results

The study cohort consisted of 13 patients with a mean age of 60.9 years (SD ± 16.8, where SD denotes standard deviation) and body mass index (BMI) of 28.1 (SD ± 8.14). The follow-up time was 53.5 months (SD ± 31.8). A total of four patients were male (30.7 %). Comorbid conditions were as follows: atrial fibrillation and chronic kidney disease in one (7 %) patient, hypertension in four (30.7 %) patents, and diabetes in two (15 %) patents. Regarding indications for surgery, prosthetic knee infection was the most common, occurring in seven (53.8 %) procedures, osteomyelitis of the tibia or femur occurred in four (30.7 %) procedures, and prosthetic hip infection and a foot infection occurred in one (7.6 %) procedure each (Table 1).

**Table 1:** Baseline demographics.

Demographic	Total group ( N=13 )
Age at surgery (years)	60.9 ± 16.8
BMI	28.1 ± 8.1
Follow-up (months)	53.5 ± 31.8
Male sex	4 (30.7 %)
Comorbidities	
Atrial fibrillation	1 (7.6 %)
Hypertension	4 (30.7 %)
Diabetes mellitus	2 (15.3 %)
Chronic kidney disease	1 (7.6 %)
Surgical indication	
Periprosthetic knee infection	7 (53.8 %)
Periprosthetic hip infection	1 (7.6 %)
Osteomyelitis of tibia or femur	4 (30.7 %)
Foot infection	1 (7.6 %)

Data measured as mean ± SD or N (%). BMI stands for body mass index.

A total of five cases received only PMMA and eight cases received PMMA with CS. No cases received exclusively CS. Overall, the mean local dosage of tobramycin was 7.6 g (range: 1.2–12.8) and the mean local dosage of linezolid was 3.2 g (range: 0.6–6) (Table 3). Nine patients (69.2 %) had no growth on preoperative cultures, two patients (15.3 %) had positive cultures for *Streptococcus agalactiae*, one patient (7.6 %) was positive for MRSA, and one patient (7.6 %) was positive for *Pseudomonas aeruginosa*. Postoperatively, nine patients (69.2 %) had no growth, three patients (23.1 %) had positive MRSA cultures, and one patient (7.6 %) had positive cultures for *Candida albicans*.

Preoperatively, patients were on oral or intravenous antibiotics based on their culture and sensitivity results. Four patients (30.7 %) were given cefazolin, three patients (23.1 %) were given clindamycin, two patients (15.3 %) were given cefepime, one patient (7.6 %) was given linezolid, and one patient (7.6 %) was given ceftriaxone, either orally or intravenously. Postoperatively, five patients (38.4 %) were prescribed linezolid, three patients (23.1 %) were given cefazolin, and cefepime and clindamycin were administered to two patients (15.3 %) each (Table 2).

**Table 2:** Systemic and local antibiotic details.

		Preoperative	Postoperative
Culture and sensitivity	MSSA	–	–
	Polymicrobial	–	–
	MRSA	1 (7.6 %)	3 (23.1 %)
	*Pseudomonas aeruginosa*	1 (7.6 %)	–
	*Streptococcus agalactiae*	2 (15.3 %)	–
	*Candida albicans*	–	1 (7.6 %)
	No growth	9 (69.2 %)	9 (69.2 %)
Systemic antibiotics	Cefazolin	4 (30.7 %)	3 (23.1 %)
	Clindamycin	3 (23.1 %)	2 (15.3 %)
	Ceftriaxone	1 (7.6 %)	–
	Cefepime	2 (15.3 %)	2 (15.3 %)
	Linezolid	1 (7.6 %)	5 (38.4 %)
	Trimethoprim–sulfamethoxazole	–	1 (7.6 %)

The abbreviations used in the table are as follows: MSSA – methicillin-sensitive *Staphylococcus aureus*; MRSA – methicillin-resistant *Staphylococcus aureus*.

**Table 3:** Local antibiotic dosage details.

Antibiotic (Carrier)	Dosage mean ± SD
Tobramycin (g) (PMMA and CS)	7.6 ± 5.1
Linezolid (g) (PMMA only)	3.2 ± 1.4

In our case series, 69.2 % ( n=9 ) of patients were successfully treated using a limb-salvage technique, which is an end-stage infection management method that is carried out to avoid amputation. The patients were infection-free after a mean follow-up of 55.5 months (range: 18–88 months); this was confirmed via clinical, radiological, and serological evaluation. In this subgroup, the average number of additional procedures that the patients underwent for eradication of infection was 2.4 (range: 1–6). Of the remaining patients, three (23.1 %) progressed to be infection-free after an above-knee amputation and one (7.7 %) was still under treatment for chronic infection suppressed on oral antibiotics at the time of writing (Table 4).

**Table 4:** Clinical outcomes.

Outcome ( N=13 )	Total ( n %)
Resolved infection and limb salvage	9 (69.2 %)
Resolved infection after above-knee amputation	3 (23.1 %)
Stable chronic infection suppressed on oral antibiotics	1 (7.7 %)

Nine patients who were successfully treated also underwent additional procedures for infection management, like primary knee arthrodesis with antibiotic cement-coated intramedullary nails (ACCIN), hindfoot fusion with ACCIN, two-stage total knee replacement arthroplasty (TKA), revision knee arthrodesis with ACCIN, single-stage TKA (with metal femur and all-polyethylene tibia components) and antibiotic cement-coated bipolar hemiarthroplasty of the hip (Table 5).

**Table 5:** Additional procedures done for infections in nine patients with resolved infection.

Procedure	Total ( n %)
Primary knee arthrodesis with ACCIN	2 (22.2 %)
Hindfoot fusion with ACCIN	2 (22.2 %)
Two-stage TKA	2 (22.2 %)
Revision knee arthrodesis with ACCIN	1 (11.1 %)
Antibiotic cement-coated bipolar hemiarthroplasty of hip	1 (11.1 %)
One-stage TKA	1 (11.1 %)

The abbreviations used in the table are as follows: TKA – total knee replacement arthroplasty; ACCIN – antibiotic cement-coated intramedullary nails.

Among the four remaining patients, three (23.1 %) required amputation due to unresolved infections, while one (7.7 %) had persistent infection and underwent primary knee arthrodesis with ACCIN, with ongoing antibiotic treatment. Two of the amputees had recurrent knee infections, undergoing prior knee arthrodesis and tissue reconstruction procedures. The last patient, medically unfit for more surgery, had two knee debridement procedures due to their cardiac issues. 

## 4 Discussion

Vancomycin is crucial in orthopedic surgery for infection prevention and treatment. Its integration into PMMA bone cement is common, but allergies pose risks to patient outcomes. PMMA, a popular carrier for antibiotic therapy, is combined with aminoglycosides, like gentamicin, tobramycin, and vancomycin. Other antimicrobials like daptomycin, linezolid, and amikacin are also utilized. PMMA spacers effectively deliver antibiotics to prevent and treat infections but are not substitutes for thorough debridement or metal implantation. Vancomycin-infused reaction (VIR) can lead to serious side effects, necessitating alternative therapies. Linezolid, effective against MRSA and vancomycin-resistant *Enterococcus* (VRE) infections, serves as an option for managing chronic and resistant orthopedic infections, particularly in patients with vancomycin allergy or resistance (Alvarez-Arango et al., 2021; Foer et al., 2023; Hepner and Castells, 2003; Symons et al., 1985; Centres for Disease Control, 2019; Courvalin, 2006).

Chiang et al. (2017) conducted a systematic literature review on the rising incidence of VRE infections in specific metropolitan areas. They found that low-risk populations have exhibited stable VRE rates since 2000. In vitro studies, including the continuous-flow chamber study of Anguita-Alonso et al. (2006), have assessed linezolid's release kinetics and activity when mixed with PMMA. Linezolid displayed the most stability, suggesting its efficiency in preventing and treating bone or joint infections. Snir et al. (2013) found that linezolid was effective for extended durations against MRSA, *S. epidermidis*, and vancomycin-resistant enterococci, especially when combined with gentamicin. Anagnostakos et al. (2008) supported this in a study on gentamicin–linezolid-loaded hip spacers for MRSA, confirming linezolid's strong antimicrobial properties, particularly in spacers without metallic components.

Nichol et al. (2016) conducted an in vitro study demonstrating that tigecycline and linezolid can be combined into bone cement without compromising their antimicrobial or biocompatibility properties, even under higher curing temperatures. Both antibiotics eluted clinically relevant concentrations within an hour and retained antimicrobial activity for a week. Wear affected tigecycline's elution but minimally impacted linezolid. The mechanical strength of linezolid-loaded cement was comparable to commercial cement, whereas tigecycline slightly reduced strength at higher concentrations. Linezolid's elution performance surpassed tigecycline's in vitro. These findings highlight linezolid's potential as an effective addition to antibiotic-loaded PMMA for orthopedic applications, particularly against MRSA.

Balato et al. (2019) found vancomycin-loaded PMMA ineffective against MRSA biofilm after 96 h, contrasting linezolid-loaded, clindamycin-loaded, and aminoglycoside-loaded cements that exhibited activity. The study highlights linezolid's bactericidal effectiveness on biofilms, emphasizing its potential advantage over vancomycin in eradicating infections due to its superior biofilm action and lower nephrotoxicity risk (Balato et al., 2019; Pritchard et al., 2010; Rybak, 2006).

Gatti et al. (2022) reviewed varies studies involving 365 patients and demonstrated linezolid's oral efficacy and safety for orthopedic implant-associated infections. The overall cure or remission rate was 79.7 %, with a discontinuation rate of up to 14.3 % due to adverse events. Linezolid, whether administered alone or with rifampicin, exhibited comparable clinical outcomes. Notably, it effectively addressed biofilm formation and achieved appropriate tissue concentrations in phase-I studies, surpassing the minimum inhibitory concentration for staphylococci and streptococci. These findings emphasize linezolid's potential in treating orthopedic implant-associated infections, indicating its efficacy against biofilm and favorable tissue concentration profiles.

The collective evidence supports linezolid's efficacy both orally and locally, with effective drug release from PMMA, presenting advantages over other antibiotics. However, further research is essential, particularly for in vivo applications in chronic bone and joint infections, MRSA cases, and instances of vancomycin allergy. In our study, linezolid-laden spacers demonstrated effectiveness in treating chronic infections in vancomycin-allergic patients. Among 13 patients, 9 (69.2 %) remained infection-free after a mean 55.5-month follow-up. Limitations include the retrospective nature of the work, lack of a comparison group, potential biases, and uncertainties with respect to off-label linezolid use in bone cement. Nevertheless, linezolid-loaded cement offers a viable management option for vancomycin-allergic patients with bone and joint infections, necessitating multicenter studies for broader efficacy assessment.

## 5 Conclusions

Enhancing bone and joint infection treatment includes exploring local antibiotic delivery, especially linezolid, as an alternative for drug-resistant pathogens and vancomycin allergies. Evidence-based guidelines for novel antibiotics are crucial. However, further research with diverse populations is needed to validate linezolid's efficacy and safety in managing infections in vancomycin-allergic patients.

## Data Availability

The data presented in this study are available upon request from the corresponding author. The data are not publicly available due to the privacy concerns regarding protected health information.
